# A Novel C-Terminal CIB2 (Calcium and Integrin Binding Protein 2) Mutation Associated with Non-Syndromic Hearing Loss in a Hispanic Family

**DOI:** 10.1371/journal.pone.0133082

**Published:** 2015-10-01

**Authors:** Kunjan Patel, Arnaud P. Giese, J. M. Grossheim, Rashima S. Hegde, Maria Delio, Joy Samanich, Saima Riazuddin, Gregory I. Frolenkov, Jinlu Cai, Zubair M. Ahmed, Bernice E. Morrow

**Affiliations:** 1 Department of Genetics, Albert Einstein College of Medicine, 1301 Morris Park Avenue, Bronx, New York, United States of America; 2 Department of Otorhinolaryngology Head & Neck Surgery, School of Medicine, University of Maryland, Baltimore, Maryland, United States of America; 3 Department of Physiology, College of Medicine, University of Kentucky, Lexington, Kentucky, United States of America; 4 Division of Developmental Biology, Cincinnati Children’s Hospital Medical Centre Cincinnati, Ohio, United States of America; 5 Department of Genetics and Genomic Sciences, Mount Sinai School of Medicine, New York, United States of America; 6 Department of Pediatrics (Clinical Genetics), Albert Einstein College of Medicine; Montefiore Medical Center, Bronx, New York, United States of America; University of Oldenburg, GERMANY

## Abstract

Hearing loss is a complex disorder caused by both genetic and environmental factors. Previously, mutations in *CIB2* have been identified as a common cause of genetic hearing loss in Pakistani and Turkish populations. Here we report a novel (c.556C>T; p.(Arg186Trp)) transition mutation in the *CIB2* gene identified through whole exome sequencing (WES) in a Caribbean Hispanic family with non-syndromic hearing loss. CIB2 belongs to the family of calcium-and integrin-binding (CIB) proteins. The carboxy-termini of CIB proteins are associated with calcium binding and intracellular signaling. The p.(Arg186Trp) mutation is localized within predicted type II PDZ binding ligand at the carboxy terminus. Our *ex vivo* studies revealed that the mutation did not alter the interactions of CIB2 with Whirlin, nor its targeting to the tips of hair cell stereocilia. However, we found that the mutation disrupts inhibition of ATP-induced Ca^2+^ responses by CIB2 in a heterologous expression system. Our findings support p.(Arg186Trp) mutation as a cause for hearing loss in this Hispanic family. In addition, it further highlights the necessity of the calcium binding property of CIB2 for normal hearing.

## Introduction

Congenital hearing loss is considered to be the most prevalent abnormality in newborns with an estimated incidence of 1/500 live births [1, 2]. Both genetic and environmental factors are responsible for hearing loss [3–6]. Genetic factors account for 50% of hearing loss in developed countries and among them the majority is non-syndromic [7]. As of June 2015, a total of 96 genes have been identified for non-syndromic hearing loss in humans (http://hereditaryhearingloss.org). Autosomal recessive non-syndromic hearing loss (ARNSHL) is the most common genetic form and accounts for ~80% of all genetic cases [8]. To date, over 60 genes have been identified for ARNSHL (http://hereditaryhearingloss.org). Previously, many ARSNHL-associated genes have been identified through linkage analysis of large consanguineous families [9]. Although this approach has been extremely successful, the requirement of large families with multiple affected and unaffected individuals has proved to be a limiting factor in linkage based gene discovery in the past.

Through recent advances in DNA sequencing technology, many previously unknown, deafness-associated genes have been discovered by use of targeted gene capture or whole exome sequencing (WES) [10–17]. This is particularly true for evaluation of smaller sized consanguineous families [18, 19] or non-consanguineous families [9]. Utilization of quartet families with one or more affected individual for WES has enabled discovery of underlying mutated genes [20–24]. The advantage of using families that are larger than trios is that the inheritance patterns can more easily aid in deciphering disease-associated mutations.

One important question in the field is whether there are differences in the relative importance of known disease genes for ARSNHL in different populations. The *GJB2* (gap junction protein beta 2) gene, comprising a single exon, mapping to the *DFNB1* locus (MIM#220290) is the most common disease-associated gene in familial or isolated ARSNHL in many populations of different ethnicities [25, 26]. We previously reported a paucity of mutations in *GJB2*, the most common cause of ARNSHL in the Bronx, NY, African-American and Caribbean Hispanic population [27–29]. Our goal to use WES was to identify mutations in other genes in individual families that are being cared for in our hospital. Here, we report results from WES of genomic DNA purified from blood, from one quartet family termed, JS6. This family is of Hispanic ethnicity that was negative for mutations in *GJB2*, and we identified a mutation in *CIB2*. Mutations in *CIB2*, encoding a calcium and integrin binding protein 2 (OMIM# 605564), were previously reported in Pakistani and Turkish families with ARSNHL (*DFNB48*) as well as Usher syndrome type 1J [30]. Through functional studies, we show here that p.Arg186Trp (C>T; chr15:78,397,660, February 2009; GRCh37/hg19 assembly) mutation specifically impaired the calcium binding ability of CIB2 in heterologous cell system.

## Materials and Methods

### Clinical Information

This research study was approved by the Albert Einstein College of Medicine Committee on Clinical Investigation (CCI#2005–756). Blood samples were collected from the JS6 family affected with hearing loss cared for at the Children’s Hospital at Montefiore, Montefiore Medical Center, NY. Family JS6 consisted of two siblings, JS6.001 (Male) and JS6.002 (Female) affected with nonsyndromic hearing loss and healthy parents, JS6.100 (mother) and JS6.200 (father). Audiometry tests were conducted in a sound proof room to evaluate hearing impairment. Different sounds of varying intensities were delivered to each ear one at a time, requiring a response from the subject thereby indicating whether or not they heard the sound. The audiologist recorded each tone at the lowest possible volume that was heard by the subject. After the general audiometry test, Rinne and Weber tuning fork tests were used to distinguish between sensorineural and conductive hearing loss. A written informed consent was attained from each individual as part of subject enrollment and before sample collection. The Puregene Genomic DNA Purification kit (Gentra, Minneapolis, MN) was used to purify DNA in the Molecular Cytogenetics Core, Albert Einstein College of Medicine, NY, according to standard protocols.

### Whole Exome Sequencing (WES)

The library preparation was performed with 1ug of genomic DNA using the Illumina TruSeq DNA Low-Throughput (LT) Sample Preparation kit (Illumina, San Diego, CA) and Roche NimbleGen SeqCap EZ Human Exome Library v3.0 according to the manufacturers instructions (Roche NimbleGen, Madison, WI). Briefly, genomic DNA was purified using the Gentra Puregene Kit (Qiagen). The quality and quantity of DNA was determined by NanoDrop spectrophotometry, Qubit fluorometery and agarose gel electrophoresis. Genomic DNA was sheared using the Covaris S2 System into 300bp fragments for DNA library preparations. Illumina TruSeq adaptors were added using the TruSeq DNA (LT) Sample Preparation kit allowing multiple samples to be barcoded simultaneously, following the protocol for ligation of adaptors. The samples were then amplified by LM-PCR following the manufacturer’s protocol (eight cycles; NimbleGen SeqCap EZ Library SR User’s Guide v3.0). The amplified libraries were analyzed for quality and quantity (Qubit).

High quality amplified sample libraries were denatured, and then hybridized to the Roche NimbleGen EZ custom design biotin-labeled Library v3.0 following the manufacturer’s protocol (NimbleGen SeqCap EZ Library SR User’s Guide v3.0). A magnetic pull down of DNA was performed with Streptavidin Dynabeads to enrich the target regions and the captured DNA was recovered. The libraries captured up to 64 Mb in a single reaction and a total of four samples were pooled per captured reaction. The captured libraries were evaluated for enrichment by real time PCR to ensure that on-target enrichment has occurred. Targeted captured enriched DNA libraries were sequenced on one lane of Illumina HiSeq 2000 instruments for paired end reads of 100 bp, as per the manufacturer’s guidelines. Images generated by the Illumina HiSeq 2000 instrument were automatically processed in real-time using control software (1.3.8) and CASAVA (1.7) software packages. Throughout this process, quality metrics were recorded to measure experimental efficacy and to facilitate rigorous filtering of sequences prior to genome alignment.

Sequencing reads were mapped to the human reference genome (GRCh37/hg19, February 2009 assembly) using BWA software (http://bio-bwa.sourceforge.net/, version 0.6.2). The PCR duplicates, to be removed, were marked using Picard-tools (http://picard.sourceforge.net, version 1.72). Local re-alignment, base quality recalibration and variant annotation were performed through GATK version 2.2–15 (Genome Analysis Toolkit, https://www.broadinstitute.org/gatk/) in UnifiedGenotyper mode. SNPs and indels were filtered using the expressions “QD < 2.0 || MQ < 40.0 || FS > 60.0 || HaplotypeScore > 13.0 || MQRankSum < -12.5 || ReadPosRankSum < -8.0” and “QD < 2.0 || ReadPosRankSum < -20.0 || FS > 200.0”, respectively. The variant call format file (VCF) for the whole exome sequence data is available at NCBI, dbGaP database, ID 16015, phs000969, title, “Whole exome sequence of hearing loss family”.

### Filtering Annotated variants

For quality control and to reduce the number of false positive calls, single nucleotide polymorphisms (SNPs) with a genotype score < 20 or a sequencing depth less < 15X were filtered and removed prior to analysis. Additionally, SNPs exceeding 10bp from either exonic boundary were also removed. The following criteria were used to prioritize the remaining variants: (1) A minor allele frequency (MAF) < = 1%, based on dbSNP (http://www.ncbi.nlm.nih.gov/projects/SNP/) and 1000 Genomes Project (http://www.1000genomes.org/); (2) A list was compiled of previously reported hearing loss genes published in the literature and listed in the Hereditary Hearing Loss database (http://hereditaryhearingloss.org/); (3) Mode of inheritance classification (using sequencing data from parents); (4) Prioritized non-synonymous variants. All remaining variants were further investigated using Alamut v2.2 software (Interactive Biosoftware, San Diego, CA). This software displays simultaneous *in silico* predictions from SIFT, Align GVGD, and MutationTaster.

### Amplification and Analysis

Prior to further investigation, all potential disease-causing variants were validated using Sanger sequencing. Primers for exon 6 of the *CIB2* gene (RefSeq: NM_006383.2 and transcript ID ENST00000258930) were designed using primer3 v0.4.0 (bioinfo.ut.ee/primer3-0.4.0/) and any SNP’s in the primer-binding site were ruled out using the NGRL SNPCheck database (https://ngrl.manchester.ac.uk/SNPCheckV3/snpcheck). PCR amplification was performed using the FASTstart High Fidelity PCR system (Roche, Madison, WI) at 59°C annealing temperature. Amplified PCR products were purified using the Agencourt AMPure XP Purification System (Beckman Coulter, Indianapolis, IN) and sequenced on the Applied Biosystems 3730 sequencer (Genomics Core at Einstein, NY). The Sequencer v4.0.1 software (Gene Codes, Ann Arbor, MI) was used to compile and compare the data to the *CIB2* sequence.

### Restriction Enzyme Digestion Assays

To determine the frequency of the c.556C>T (p.(Arg186Trp)) *CIB2* mutation in the healthy Caribbean Hispanic population, a PCR product based restriction enzyme digestion assay was developed. We selected 194 healthy controls and 94 of which were ethnically matched (Caribbean Hispanic). The HPAII enzyme (New England Biolabs, Ipswich, MA) that recognizes the 5’ CCGG 3’ restriction site, was, abolished by the c.556C>T mutation of *CIB2*. In our assay, any samples with c.556C>T mutation will remain undigested (206 bp) whereas the digestion of a normal sample would result into two restriction fragments 124 bp and 82 bp size. Any heterozygous or homozygous samples identified through the restriction enzyme digest assay would be sequenced for confirmation.

PCR amplification was performed using the FASTstart High Fidelity PCR system (Roche, Madison, WI) at 60°C annealing temperature. Amplified products where then digested for 16 hours at 37°C. Digested PCR products were then run on a 2% agarose gel long with a 100bp sizing ladder.

### DNA constructs

The full-length isoform of human *CIB2* was PCR amplified from adult human eye cDNA (Clontech, Mountain View, CA), cloned into the pEGFP-N2 vector and sequence was verified. Stratagene QuikChange Lightning mutagenesis (Roche) was used to introduce the c.556C>T (Fwd-primer 5’-cctcagcactttccacatctggatccccgggatcc-3’; Rev-primer 5’ ggatcccggggatccagatgtggaaagtgctgagg-3’) mutation into the wild type *CIB2* sequence.

### Helios gene gun transfection

Postnatal day 3 (P3) vestibular sensory epithelial explants from C57BL/6 mice were cultured for 24h in DMEM supplemented with 10% FBS (Life Technologies, Carlsbad, CA) at 37°C with 5% CO_2_. Explants were transfected with constructs encoding CIB2^WT^-GFP, and CIB2^R186W^-GFP using a Helios gene gun. After 24h, cells were fixed in 4% paraformaldehyde and counter-stained with rhodamine phalloidin and DAPI (Invitrogen). Finally, samples were mounted with the ProLong Gold Reagent and imaged using a 100X objective and a confocal microscope (LSM700, Carl Zeiss).

### Immunostaining of COS-7 cells

COS-7 (African green monkey fibroblast cell line; ATCC CRL-1651; [31]) cells were co-transfected with 2μg of GFP-Myosin 15a, Dsred-CIB2^WT^, Dsred-CIB2^R186W^ and Whirlin constructs using Lipofectamine-2000 (Life Technologies, Carlsbad, CA). After 24h of transfection, COS-7 cells were trypsinized and plated on 35mm glass-bottom dishes (MatTek, Ashland, Maine). COS-7 cells were fixed with 4% PFA after 24h and processed for immunostaining. Whirlin antibodies (HL5141; [32]) were used at 1/500 dilution and incubated overnight at 4°C, followed by washing and secondary antibody labeling with AlexaFluor 647 goat anti rabbit antibodies. COS-7 cells were mounted using the prolong gold antifade reagent and imaged using a 63X objective and a confocal microscope (LSM700, Carl Zeiss).

### Co-immunoprecipitation assay

HEK 293 cells [33] were maintained using DMEM supplemented with 10% FBS, glutamine and penicillin-streptomycin (Invitrogen). Cells were plated in 100mm culture dishes for 24h at 37°C in 5% CO_2_. On the day of transfection, 10μg of each DNA sample was transfected into cells using Polyethylenimine (PEI; Polysciences). After 48h, cells were washed with cold 1x PBS, then homogenized with a sonicator (Fisher Scientific) at intensity setting 2 for 10s in RIPA buffer containing a protease inhibitor mixture (Roche). Protein A–Sepharose CL-4B beads were incubated for 4h with 5 μg of antibody to GFP (Life Technologies) and were washed three times with PBS containing 0.1% Triton X-100. Lysates were incubated with the beads overnight at 4°C and were centrifuged at 10,000*g* for 3m. Beads were washed with RIPA buffer three times and boiled in 2× SDS sample buffer. Samples were processed for western blot using 4–20% Tris Glycine gel (Novex) as well as antibodies against GFP and DsRed (Clontech) tags.

### Calcium imaging

The calcium imaging was done as previously described [30]. Briefly, HEK-293 cells were transfected with 3–4 ug of DsRed-tagged *CIB2* constructs using Lipofectamine 2000 (Life Technologies, Carlsbad, CA). After 24h, cells were loaded with 18 μM ratiometric Ca^2+^ indicator, Fura-2 AM (Life Technologies, Carlsbad, CA), for 1–1.5h at room temperature. Fura-2 fluorescence was observed in L-15 medium at room temperature with sequential 340- and 380-nm illuminations at a rate of 0.78–0.81 image pairs per second. The 340- to 380-nm fluorescence (F340/F380) ratio images were calculated, and pixel values were converted to intracellular Ca^2+^ concentration using the calibration curve obtained with the Fura-2 Calibration kit (Life Technologies, Carlsbad, CA). Calcium responses were evoked by application of 1 μM ATP for 50 s through a puff pipette of ~1 μm in diameter that was situated ~25 μm from the cells. The number of dishes used for every construct was 4 or greater, and the number of transfected cells for every construct was over 40. The data was analyzed by a student *t*-test and P values <0.05 were considered statistically significant.

### Molecular modeling

CIB2 was modeled using the high-resolution crystal structure of human CIB1 (PDB 1XO5) as a template and the SWISS-MODEL server [34]. Energy minimization and analysis were performed with a Yasara server [35].

## Results

### Affected individuals of Hispanic family have nonsyndromic hearing loss

**Fig 1:**
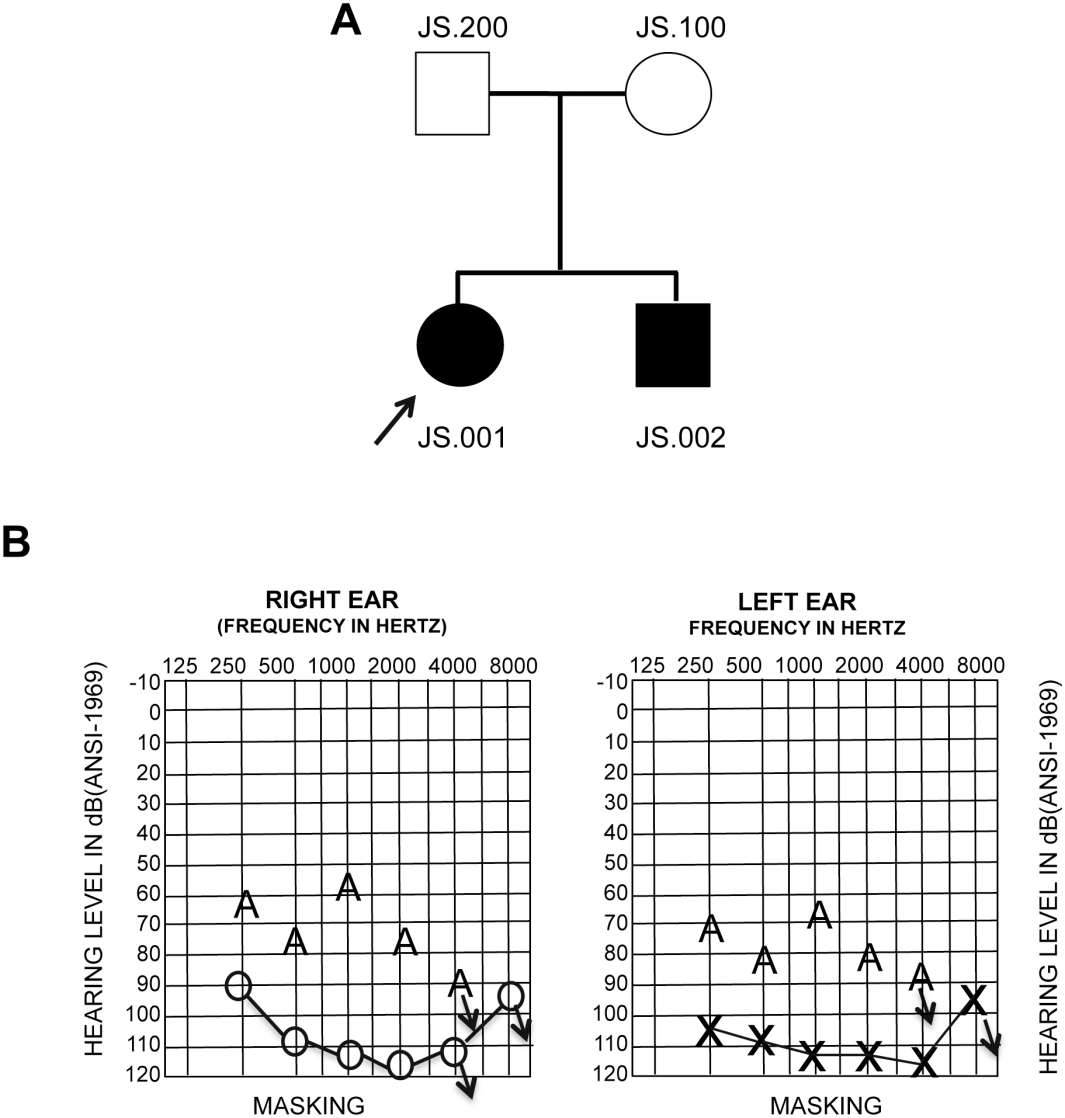
Pedigree and audiogram of JS6 proband. (A) Pedigree of family JS6 (arrow to proband). The filled symbols represent affected individuals. (B) Audiogram from the female proband, JS6.001 indicating hearing loss ranging from severe to profound. The symbols ‘o’ and ‘x’ denote air conduction pure-tone thresholds, and the ‘A’ symbol denotes bone conduction thresholds. Downward arrow denotes no response on the audiogram.

We identified a Caribbean Hispanic family in which both affected children have bilateral profound hearing loss diagnosed within the first year of life (Fig 1A). Additional family history was obtained through a medical interview at the time of recruitment. The parents were found to have normal hearing and did not report any medical complication or drug exposure during pregnancy. No evidence for other syndromic features such as Usher syndrome was present. The proband had a normal ocular exam (Ocular exam: VA: 20/25 left, 20/20 right; EOM full. External: OK; Anterior segment: L/L flat on; FUNDUS: = 0.75–1.00 x 180; +2.50–1.50 x 180). There was no evidence for congenital heart disease, respiratory problems, urinary tract abnormalities, thereby ruling out the possibility of CHARGE syndrome. Assessment of auditory brainstem responses (ABR) at 7 months revealed profound impairment with a threshold greater than 90 dB at 250 Hz and greater than 110 dB for all other frequencies tested (Fig 1B). The acoustic reflexes were also absent in both ears. The proband’s brother had bilateral hearing loss (Fig 1A), but there is no other family history of hearing loss. Therefore, based on this information we tentatively concluded that hearing loss in both children is non-syndromic with a possible underlying genetic cause. Mutations in the *GJB2* gene had previously been excluded as part of routine clinical management.

### WES revealed a missense mutation in *CIB2* associated with nonsyndromic hearing loss

WES analysis was performed on DNA from family JS6, including both affected children and parents. WES generated an average of 7.5 billion base pair sequence, with an average map ability rate of 95% across the 64Mb targeted region. The mean target depth for these samples was approximately 57X. This data resulted in a total of 36,752 single nucleotide variants (SNVs) and 21,721 indels after variant calling using GATK. The data was then compared to the dbSNP and 1000 Genomes Project databases to eliminate SNVs with a MAF >1%. The SNVs were further categorized based on the mode of inheritance using the parental sequencing data. As a result, we obtained 619 recessively inherited variants of which 535 were SNVs and 84 were indels. This group also included compound heterozygous DNA variants and variants, which were homozygous in either affected child. A total of 53 SNVs were identified in both affected individuals and they were analyzed first. We prioritized our analysis using a custom list of 160 genes associated with both syndromic and non-syndromic hearing loss. We included both types of hearing loss because mutations in genes for syndromic hearing loss have been found in non-syndromic individuals. The 160 genes were complied through literature search and presence in the Hereditary Hearing Loss database (http://hereditaryhearingloss.org/).

**Fig 2:**
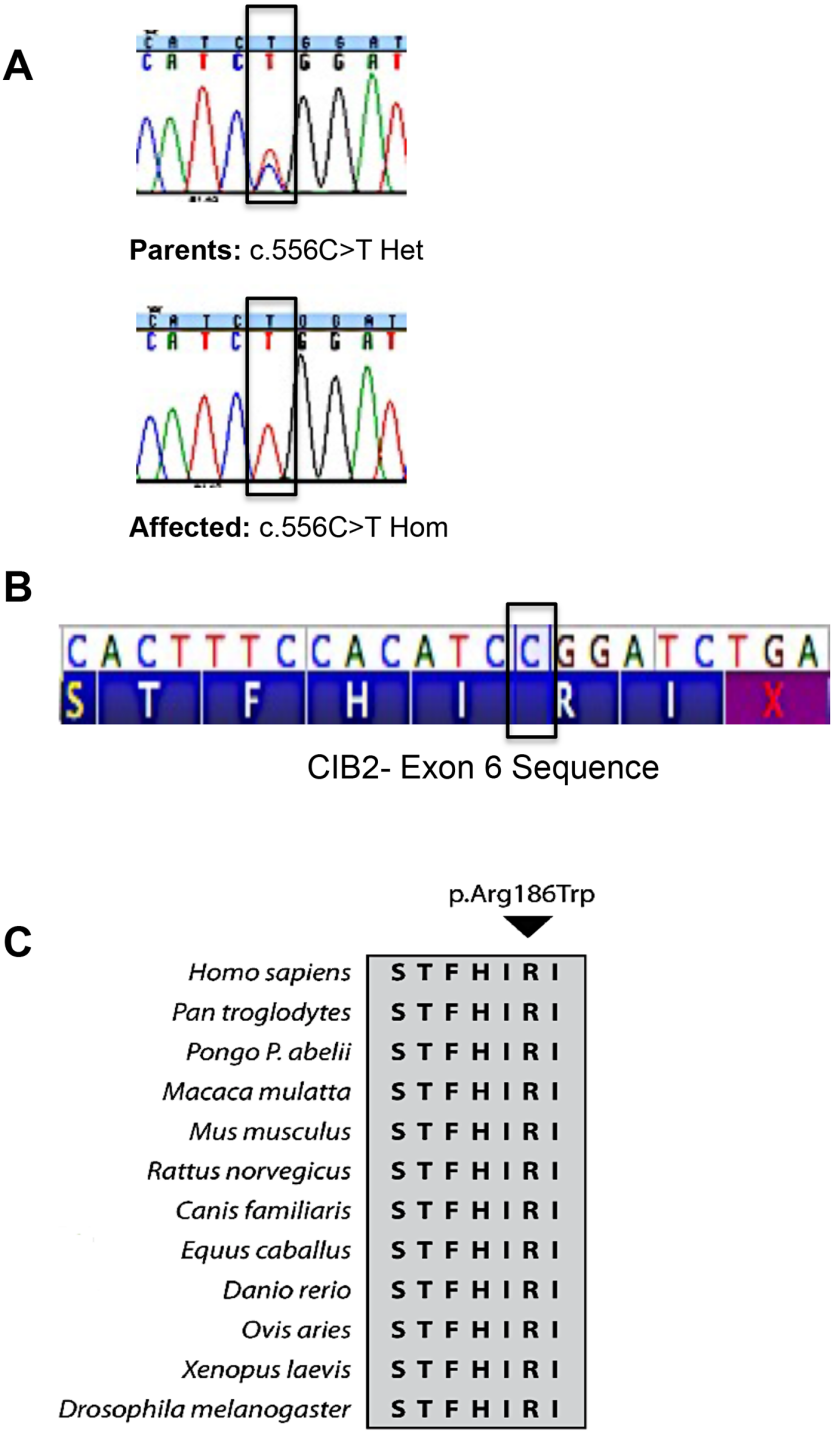
Mutation in penultimate amino acid of *CIB2*. (A) Chromatogram from Sanger sequencing showing that both affected children have a homozygous c.556C>T (p.Arg186Trp) mutation and the parents are heterozygous carriers. The chromatogram of the proband is shown and the sibling has an identical chromatogram (not shown). (B) The mutation affects the arginine residue at amino acid position 186 of CIB2, which is the penultimate amino acid of the protein. The genomic position of the nucleotide variant is on chromosome 15, position 78,397,660 on human February 2009, GRCh37/hg19 assembly. (C) The arginine residue at amino acid position 186 is conserved across a wide variety of species. Identical residues are highlighted in gray color.

**Fig 3:**
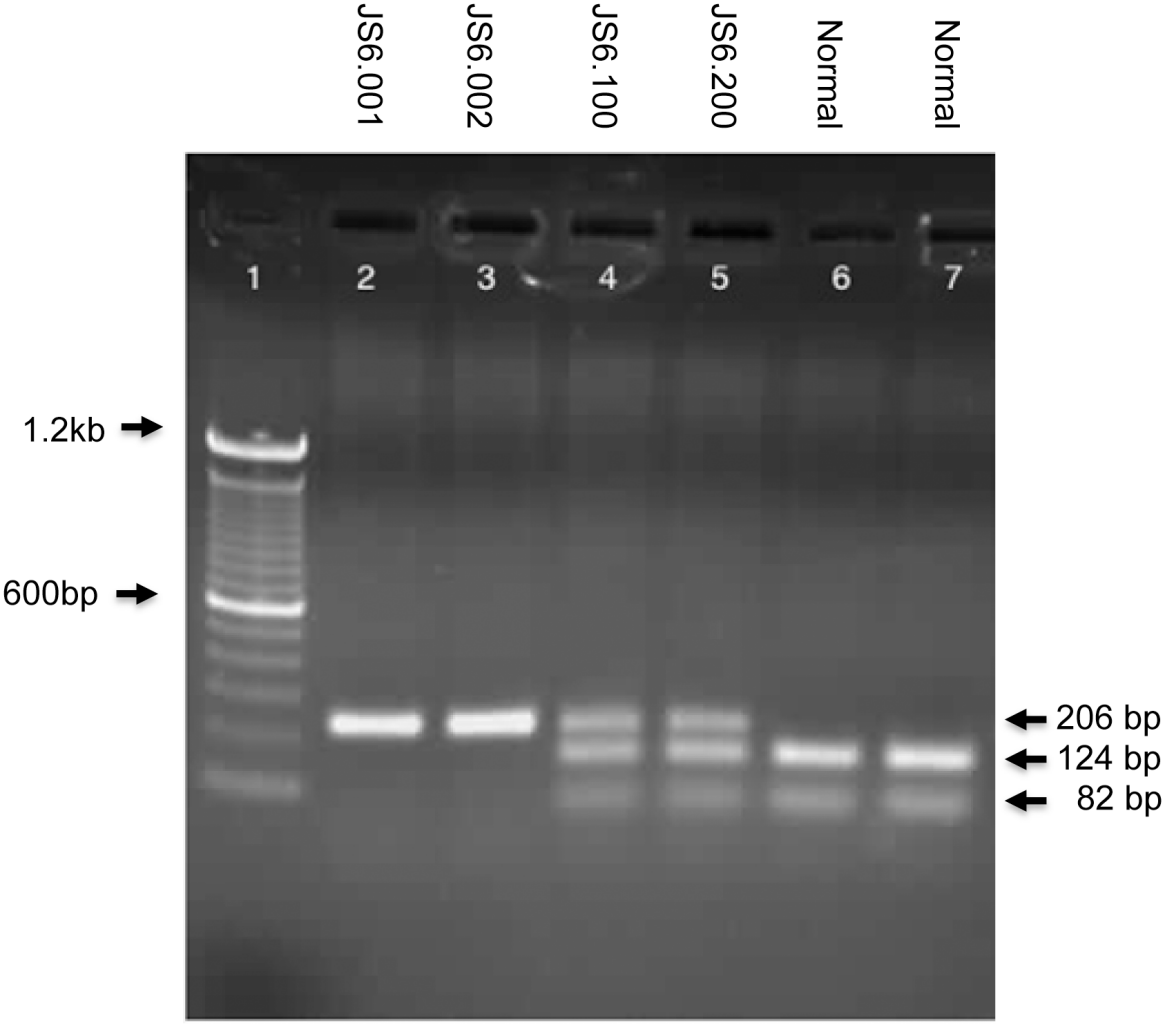
Restriction enzyme digestion to validate the *CIB2* mutation. Lanes 2 and 3 on the agarose gel represent the restriction digest of a PCR product that was performed on both affected children. The presence of the c.556C>T mutation abolishes the restriction site and results in a single product of 206bp (Lanes 2 and 3, depict the proband and sibling, respectively). Lanes 4 and 5 contain both parental samples and as a result the there is a PCR product of 206bp representing the mutant allele as well as two additional digested fragments at 124bp and 82bp, which represent the normal allele. Lanes 6 and 7 are restriction enzyme digests from two normal, unrelated individuals, with no PCR product corresponding to the mutant allele of 206bp and only two digested PCR products corresponding to the normal allele. Lane 1 contains the DNA size standard ladder.

Through this initial search, we identified a homozygous mutation in *CIB2* (c.556C>T; p.(Arg186Trp)) in both affected siblings, whereas the healthy parents are unaffected carriers (Fig 2). The c.556C>T (p.Arg186Trp) mutation is located in exon 6 of *CIB2*, which encodes the carboxy-terminal end of the resulting polypeptide. The mutation in the female proband found by WES was validated by Sanger sequence analysis (Fig 2A). This nucleotide change was not observed in the normal population (1000 Genomes Project; NHLBI ESP6500). We performed a mutation specific restriction enzyme digest (Fig 3) in 94 ethnically matched (Hispanic) and a 100 African American unrelated healthy individuals and did not identify the mutation in any of the subjects, suggesting this mutation is truly rare in both populations (data not shown). This nucleotide change has been observed as a rare heterozygous variant in an African American control samples and was absent in a further 8586 European controls samples studied, as part of the NHLBI Exome Sequencing Project (ESP; S1 Table).

The arginine residue at position 186 (Fig 2B) is located in a predicted type II PDZ binding ligand (aa184-187,-HIRI-COO^-^, X Φ_1_ X Φ_2_-COO^-^). It is the penultimate amino acid of the protein (Fig 2B). This residue shows moderate evolutionary conservation, through twelve mammalian species and a moderate Grantham distance between the arginine and tyrosine (101) as depicted in Fig 2C. Evolutionary conservation between species would suggest that this particular amino acid might be important for the function of the protein. Structural differences in amino acid substitutions could influence the function as well. *In silico* analysis using Align DVGD, CADD, SIFT and MutationTaster predicted this mutation to be damaging (S1 Table).

We examined WES data for other possible candidate genes as well. We identified an intronic variant in *CHD7* (g.61713126_61713127insTGGACT; Human February 2009 GRCh37/hg19 Assembly). Heterozygous mutations in this gene have been associated with CHARGE syndrome. CHARGE syndrome is a multi-systemic disorder including hearing loss in a subset of patients. Based upon the medical history, there was no evidence for clinical features of CHARGE syndrome, including congenital heart disease, respiratory problems or urinary tract abnormalities, thereby ruling out this variant as being responsible for deafness in this family. No other mutations, in any of the 160 known hearing loss causing genes, were identified.

### Targeting of CIB2 to the tips of stereocilia and CIB2-Whirlin-Myosin 15a complex was not affected by p.Arg186Trp mutation

**Fig 4:**
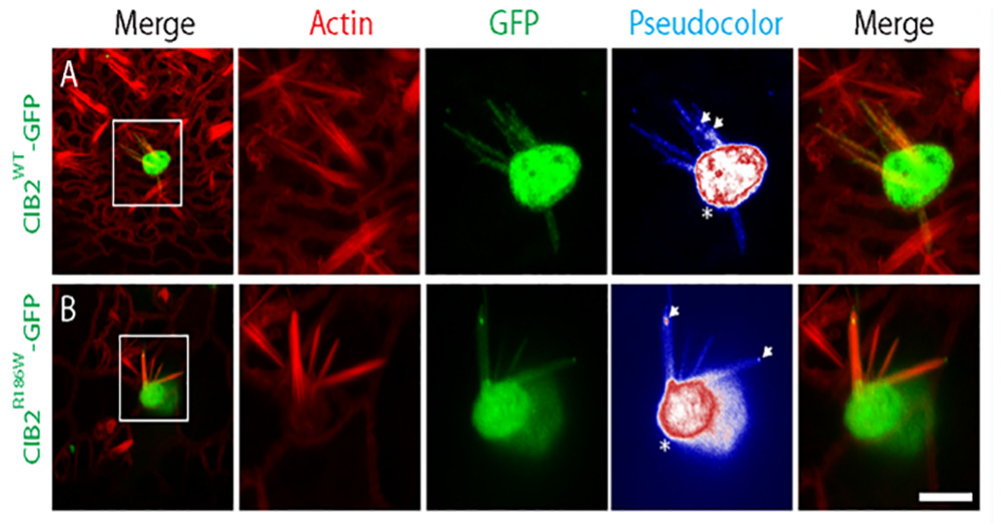
The p.Arg186Trp mutation does not affect the targeting of CIB2 to the stereocilia tips of vestibular system hair cells. Gene gun transfection of P3 vestibular system with a CIB2^WT^-GFP expression vector shows targeting of CIB2 to the cell body, the cuticular plate (Pseudocolor, *) and also along the length of stereocilia of hair cells (top set of panels). As previously shown, CIB2 also accumulates to the stereocilia tips (Pseudocolor, arrows). The p.Arg186Trp mutation does not affect the localization of CIB2 in the cuticular plate or to the tip of stereocilia (pseudocolor, *, arrows) as shown in the bottom set of panels. Scale bars, 5μm.

CIB2 is localized to the stereocilia of cochlear and vestibular system hair cells, primarily at the tip of stereocilia of the shortest rows in hair cells, where the mechanotransduction channels are localized [30]. To investigate the effect of p.Arg186Trp mutation on CIB2 targeting to the tip of stereocilia, we overexpressed GFP tagged CIB2^R186W^ protein in postnatal vestibular system explants, using a Helios gene gun mediated transfection system. The targeting of CIB2^R186W^-GFP was comparable to CIB2^WT^-GFP (Fig 4). These results suggest that in explant cultures, the p.Arg186Trp alleles do not affect the targeting of CIB2 to the tip of stereocilia.

**Fig 5:**
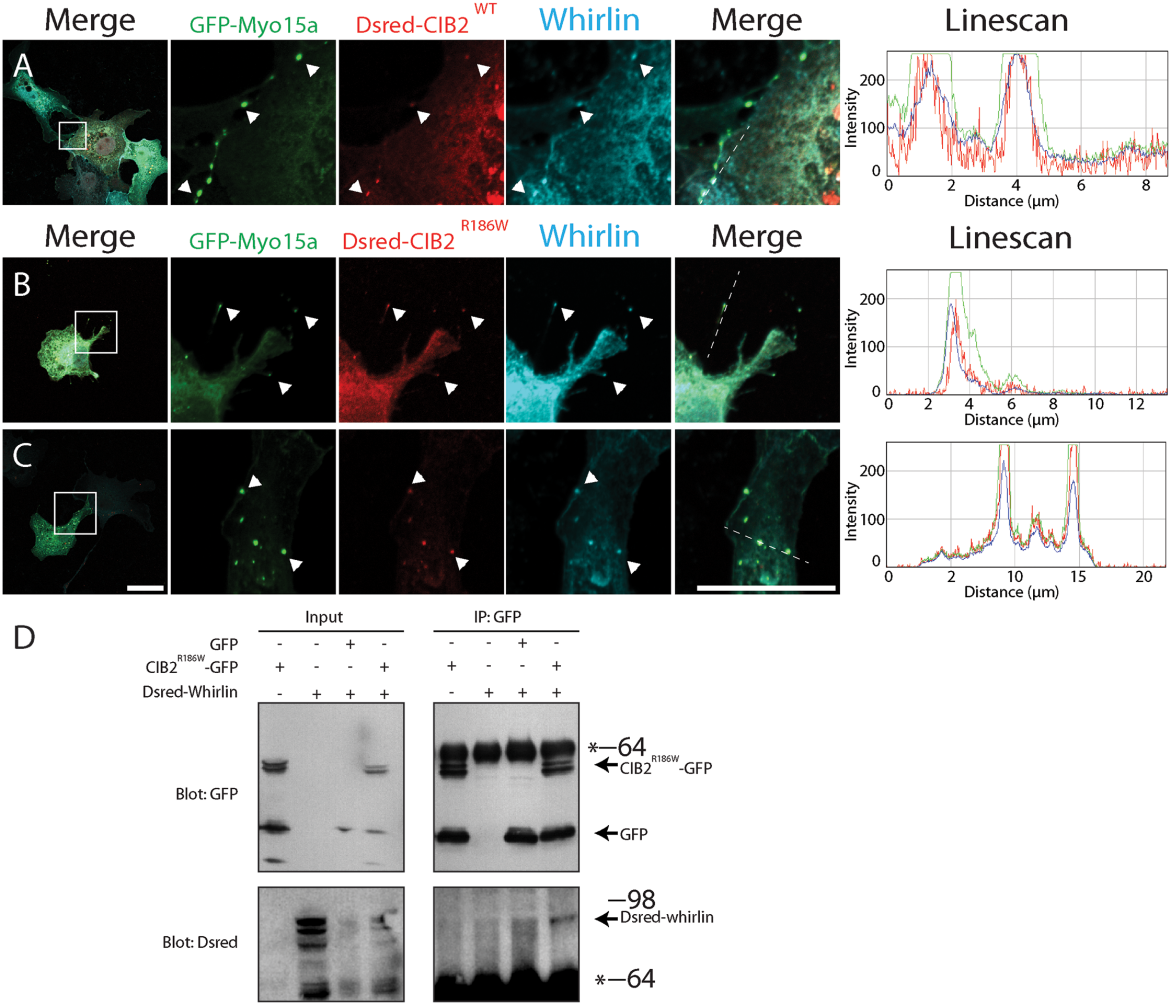
The p.Arg186Trp mutation does not affect the Myosin 15a/Whirlin/CIB2 tripartite complex. COS-7 cells were co-transfected with GFP-Myosin 15a, Dsred-CIB2^WT^, Dsred-CIB2^R186W^ and Whirlin constructs. A) Co-transfection of GFP-Myosin 15a, Dsred-CIB2^WT^ and Whirlin shows that the Myosin 15a-Whirlin complex is able to transport CIB2 to the tip of the filopodia and form a tripartite complex. The linescan analysis shows co-localization of the three proteins. B, C) The p.Arg186Trp mutation does not affect transport of CIB2 as Dsred-CIB2^R186W^ co-localizes with Whirlin and Myosin 15a at the tip of the filopodia. D) *In vitro* co-immunoprecipitation of CIB2^R186W^-GFP and Dsred-Whirlin constructs showing that CIB2^R186W^ variant interacts with Whirlin. GFP construct is used as a negative control. Scale bars, 10μm.

We previous have shown that CIB2, Whirlin and Myosin XVa forms a tripartite complex located at the tip of filopodia of COS-7 cells [30]. To determine if the deafness causing mutation affects the ability to localize to the tips of filopodia, we over-expressed DsRed tagged CIB2^WT^ (Fig 5A) and CIB2^R186W^ constructs of human *CIB2* (Fig 5B and 5C) along with non-tagged human Whirlin and GFP-Myosin 15a in COS-7 cells. Confocal imaging of transfected COS-7 cells revealed that CIB2 and CIB2^R186W^ variants are located at the tip of filopodia (Fig 5), indicating the persistence of the interaction between CIB2 and Whirlin despite the presence of the mutation. The interaction between CIB2^R186W^ variant and Whirlin was further confirmed by *in vitro* co-immunoprecipitation assay (Fig 5D).

### The p.Arg186Trp mutation affects the calcium binding affinity of CIB2

**Fig 6:**
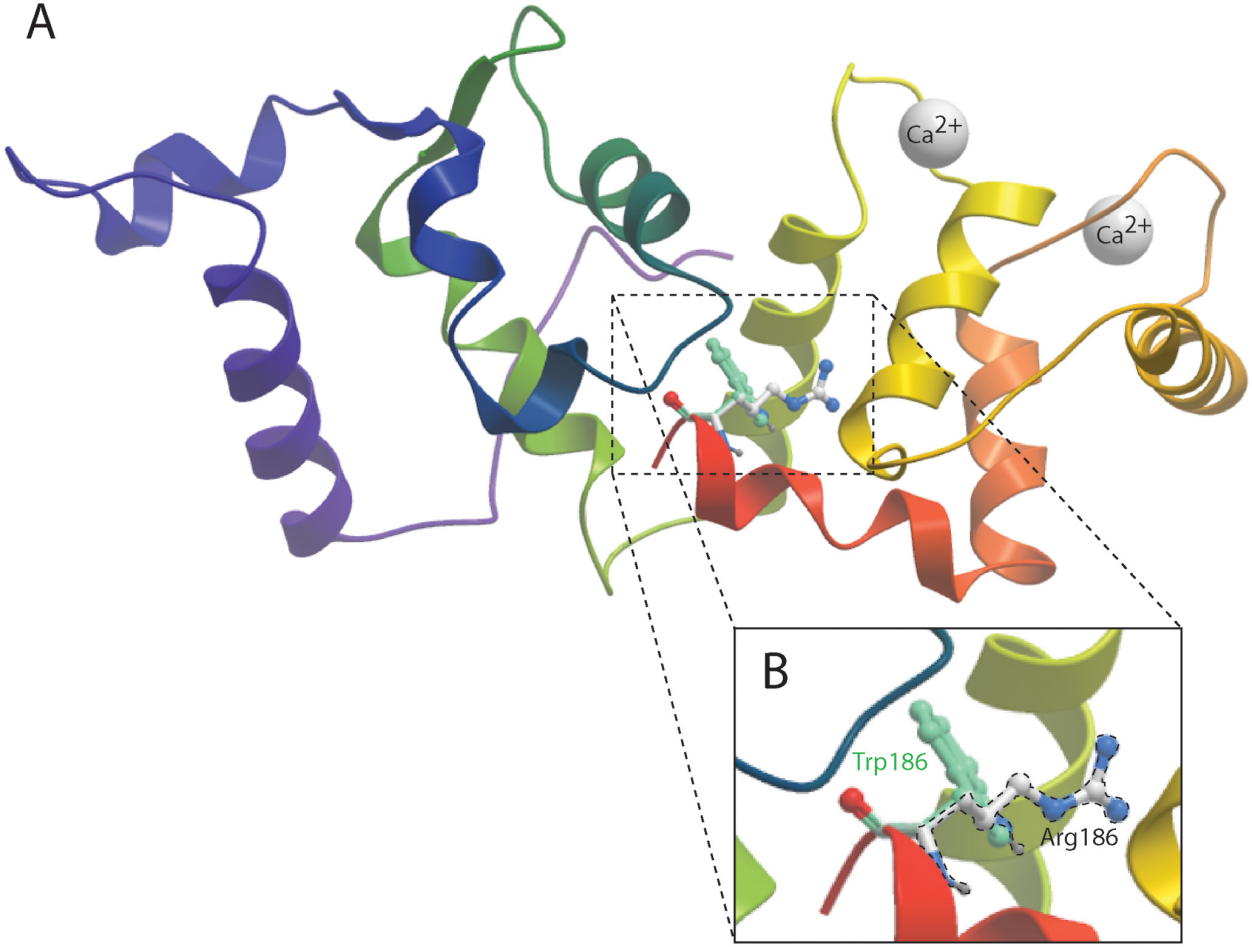
The C-terminal helix of CIB2 mutation may be destabilized because of steric hindrance. Molecular models using the Protein Data Bank (PDB) 1XO5 crystal structure of Ca^2+^-CIB1 as a template. A) The backbone ribbon of the C-terminal helix of CIB1 is highlighted in red, and the four Ca^2+^ ions are represented by white spheres. B) The side-chain of the Arg186 residue is represented in white and blue (dash line), and the Trp residue is overlapped in green at position 186.

**Fig 7:**
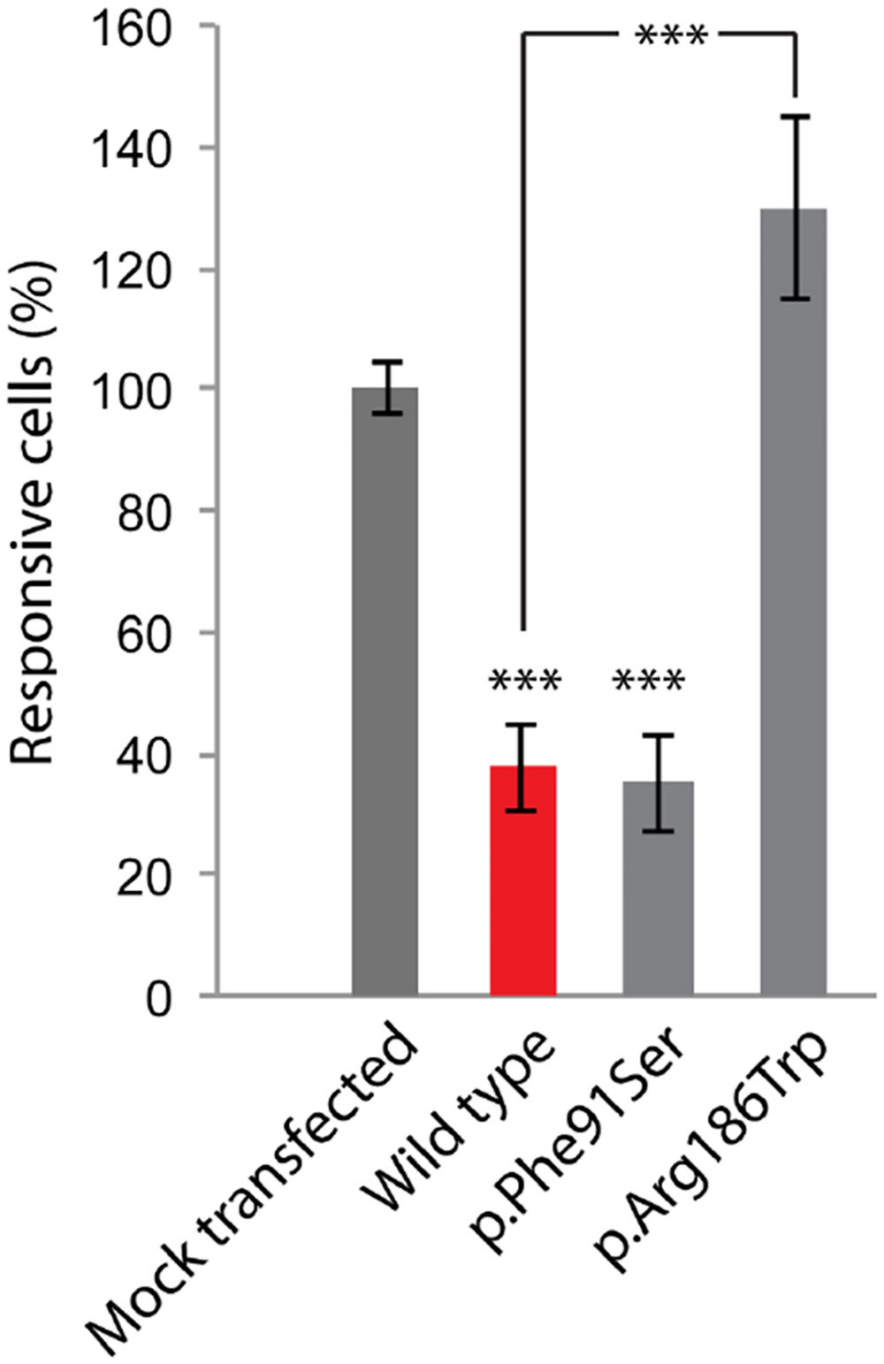
The p.Arg186Trp mutation affects the calcium binding affinity of CIB2. Calcium responses in COS-7 cells transfected with DsRed-tagged CIB2 constructs were recorded after ATP stimulation. The p.Arg186Trp mutation abolished the ability of CIB2 to decrease ATP-induced calcium release from the cell, whereas the p.Phe91Ser mutation did not. Data are normalized to the average response of mock-transfected cells and are shown as mean ± s.e.m. ***P < 0.001; **P < 0.01; *P < 0.05.

We analyzed the effect of the p.Arg186Trp mutation on the molecular structure of CIB2, using the human crystal structure of CIB1 (1XO5) as a template. The carboxy terminal helix of CIB1 is flexible and believed to participate in the Ca^2+^ binding. This helix is folded back against the hydrophobic integrin binding pocket of the CIB1, C-domain in the 1XO5.PDB crystal structure. The displacement of this helix is also part of the integrin binding mechanism suggesting that it might affect access to the hydrophobic pocket to integrins. Any unfolding or destabilization of the helix due to p.Arg186Trp allele could potentially affect the ability for CIB2 to bind to calcium or integrins (Fig 6). To test if the pArg186Trp mutation affects the calcium binding affinity of CIB2, we measured the inositol triphosphate (IP3)-dependent Ca^2+^ responses evoked by extracellular ATP in HEK-293 cells transiently transfected with Dsred tagged constructs (Fig 7). As a control we measured responses in the cells expressing the p.Phe91Ser variant of CIB2 [30]. HEK-293 cells overexpressing wild type CIB2 demonstrated significantly decreased ATP-induced Ca^2+^ responses as compared to no Ca2+ buffering ability in mock transfected cells, as previously reported [30]. Cells transfected with the p.Phe91Ser mutant allele did not alter the calcium binding affinity of CIB2 (Fig 7). Intriguingly, HEK-293 cells overexpressing p.Arg186Trp mutant CIB2 had a significant increase in Ca^2+^ responses, indicating that this CIB2^R186W^ variant resulted in loss of Ca2+ sequestering ability (Fig 7).

## Discussion

Using WES, we have identified a homozygous *CIB2* mutation in a family with non-syndromic sensorineural hearing loss. The p.Arg186Trp mutation is located within the carboxy terminal end of the CIB2 protein and is predicted to be pathogenic by *in silico* prediction databases (S1 Table). CIB2 belongs to the family of calcium-and integrin-binding proteins, consisting of three additional CIB family members (CIB 1, 3 and 4) [36]. These proteins are characterized by three to four EF hand motifs and are involved in mediating Ca2+ binding and intracellular signaling [37].

In the sensory epithelium of the inner ear, CIB2 is targeted to the tip of the stereocilia of hair cells, a site of the tip-link mechanotransduction complex [38–41]. There are several important proteins that comprise the complex, including Protocadherin 15 and Cadherin 23, which form the extracellular tip link structure and are mutated in patients with Usher type 1 syndrome. The tip link is connected inside the cell by Whirlin and Myosin VIIa, as well as other proteins, which are also mutated in patients with ARSNHL [42]. Biochemical studies demonstrated that CIB2 functionally and physically interacts with Whirlin and Myosin VIIa [30]. We found that the p.Arg186Trp mutation does not affect the tip localization as well as interaction between CIB2 and Whirlin. However, we cannot exclude the possibility that CIB2 harboring p.Arg186Trp mutation dimerizes with endogenous CIB2 and that this dimer is targeted to the tip of stereocilia. CIB2 belongs to the calcium integrin binding family and shares significant sequence homology with CIB1, which has been extensively studied and is known to regulate platelet aggregation by interacting with platelet integrin alpha(IIb)beta(3). However, the Arg186 residue is not conserved between CIB1 and CIB2. CIB1 specifically binds to platelet integrin alpha(IIb)beta(3) through displacement of its C-terminal end [43]. In contrast, our *ex vivo* functional studies revealed that the p.Arg186Trp mutation within the carboxy terminus of CIB2 affects the calcium binding ability. Our study indicates that the mutations of *CIB2* are not limited to Pakistanis or Turkish populations. Detection of p.Arg186Trp allele in Caribbean Hispanic family and in an African American control sample (S1 Table) raises the probability that individuals of other populations may harbor mutations in *CIB2*.

## Supporting Information

S1 Table In silico analysis of the CIB2: c.556C>T (p.Arg186Trp) mutation. We performed a bioinformatic analysis of the c.556C>T mutation in CIB2 to determine its predicted pathogenicity. We provide the SNP ID number from dbSNP, frequency of occurrence in European and African American populations as well as results from pathogenicity predication software.
(DOCX)

## References

[1] Morton CC, Nance WE. Newborn hearing screening—a silent revolution. The New England journal of medicine. 2006;354(20):2151–64. . 16707752 10.1056/NEJMra050700

[2] Marazita ML, Ploughman LM, Rawlings B, Remington E, Arnos KS, Nance WE. Genetic epidemiological studies of early-onset deafness in the U.S. school-age population. American journal of medical genetics. 1993;46(5):486–91. 10.1002/ajmg.1320460504 . 8322805

[3] Friedman TB, Griffith AJ. Human nonsyndromic sensorineural deafness. Annual review of genomics and human genetics. 2003;4:341–402. 10.1146/annurev.genom.4.070802.110347 . 14527306

[4] Duman D, Tekin M. Autosomal recessive nonsyndromic deafness genes: a review. Frontiers in bioscience. 2012;17:2213–36. 22652773 10.2741/4046 PMC3683827

[5] Cohen BE, Durstenfeld A, Roehm PC. Viral causes of hearing loss: a review for hearing health professionals. Trends in hearing. 2014;18 10.1177/2331216514541361 25080364 PMC4222184

[6] Deltenre P, Van Maldergem L. Hearing loss and deafness in the pediatric population: causes, diagnosis, and rehabilitation. Handbook of clinical neurology. 2013;113:1527–38. 10.1016/B978-0-444-59565-2.00023-X . 23622376

[7] Cremers CW, Marres HA, van Rijn PM. Nonsyndromal profound genetic deafness in childhood. Annals of the New York Academy of Sciences. 1991;630:191–6. . 1952589 10.1111/j.1749-6632.1991.tb19587.x

[8] Maheshwari M, Vijaya R, Ghosh M, Shastri S, Kabra M, Menon PS. Screening of families with autosomal recessive non-syndromic hearing impairment (ARNSHI) for mutations in GJB2 gene: Indian scenario. American journal of medical genetics Part A. 2003;120A(2):180–4. . 12833397 10.1002/ajmg.a.20014

[9] Najmabadi H, Kahrizi K. Genetics of non-syndromic hearing loss in the Middle East. International journal of pediatric otorhinolaryngology. 2014;78(12):2026–36. 10.1016/j.ijporl.2014.08.036 . 25281338

[10] Avraham KB, Kanaan M. Genomic advances for gene discovery in hereditary hearing loss. Journal of basic and clinical physiology and pharmacology. 2012;23(3):93–7. 10.1515/jbcpp-2012-0033 . 22962211

[11] Bamshad MJ, Ng SB, Bigham AW, Tabor HK, Emond MJ, Nickerson DA, et al Exome sequencing as a tool for Mendelian disease gene discovery. Nature reviews Genetics. 2011;12(11):745–55. 10.1038/nrg3031 . 21946919

[12] Shearer AE, Smith RJ. Genetics: advances in genetic testing for deafness. Current opinion in pediatrics. 2012;24(6):679–86. 10.1097/MOP.0b013e3283588f5e 23042251 PMC3694178

[13] Zhang X. Exome sequencing greatly expedites the progressive research of Mendelian diseases. Frontiers of medicine. 2014;8(1):42–57. 10.1007/s11684-014-0303-9 . 24384736

[14] Kim NK, Kim AR, Park KT, Kim SY, Kim MY, Nam JY, et al Whole-exome sequencing reveals diverse modes of inheritance in sporadic mild to moderate sensorineural hearing loss in a pediatric population. Genetics in medicine: official journal of the American College of Medical Genetics. 2015 10.1038/gim.2014.213 . 25719458

[15] Atik T, Bademci G, Diaz-Horta O, Blanton SH, Tekin M. Whole-exome sequencing and its impact in hereditary hearing loss. Genetics research. 2015;97:e4 10.1017/S001667231500004X . 25825321 PMC5503681

[16] Haraksingh RR, Jahanbani F, Rodriguez-Paris J, Gelernter J, Nadeau KC, Oghalai JS, et al Exome sequencing and genome-wide copy number variant mapping reveal novel associations with sensorineural hereditary hearing loss. BMC genomics. 2014;15:1155 10.1186/1471-2164-15-1155 . 25528277 PMC4367882

[17] Rehman AU, Santos-Cortez RL, Morell RJ, Drummond MC, Ito T, Lee K, et al Mutations in TBC1D24, a gene associated with epilepsy, also cause nonsyndromic deafness DFNB86. American journal of human genetics. 2014;94(1):144–52. 10.1016/j.ajhg.2013.12.004 24387994 PMC3882911

[18] Shamseldin HE, Tulbah M, Kurdi W, Nemer M, Alsahan N, Al Mardawi E, et al Identification of embryonic lethal genes in humans by autozygosity mapping and exome sequencing in consanguineous families. Genome biology. 2015;16(1):116 10.1186/s13059-015-0681-6 . 26036949 PMC4491988

[19] Makrythanasis P, Nelis M, Santoni FA, Guipponi M, Vannier A, Bena F, et al Diagnostic exome sequencing to elucidate the genetic basis of likely recessive disorders in consanguineous families. Human mutation. 2014;35(10):1203–10. 10.1002/humu.22617 . 25044680

[20] Glazov EA, Zankl A, Donskoi M, Kenna TJ, Thomas GP, Clark GR, et al Whole-exome re-sequencing in a family quartet identifies POP1 mutations as the cause of a novel skeletal dysplasia. PLoS genetics. 2011;7(3):e1002027 10.1371/journal.pgen.1002027 21455487 PMC3063761

[21] Yuen RK, Thiruvahindrapuram B, Merico D, Walker S, Tammimies K, Hoang N, et al Whole-genome sequencing of quartet families with autism spectrum disorder. Nature medicine. 2015;21(2):185–91. 10.1038/nm.3792 . 25621899

[22] Okou DT, Mondal K, Faubion WA, Kobrynski LJ, Denson LA, Mulle JG, et al Exome sequencing identifies a novel FOXP3 mutation in a 2-generation family with inflammatory bowel disease. Journal of pediatric gastroenterology and nutrition. 2014;58(5):561–8. 10.1097/MPG.0000000000000302 24792626 PMC4277865

[23] Qing J, Yan D, Zhou Y, Liu Q, Wu W, Xiao Z, et al Whole-exome sequencing to decipher the genetic heterogeneity of hearing loss in a Chinese family with deaf by deaf mating. PloS one. 2014;9(10):e109178 10.1371/journal.pone.0109178 25289672 PMC4188603

[24] Yang Y, Muzny DM, Reid JG, Bainbridge MN, Willis A, Ward PA, et al Clinical whole-exome sequencing for the diagnosis of mendelian disorders. The New England journal of medicine. 2013;369(16):1502–11. 10.1056/NEJMoa1306555 24088041 PMC4211433

[25] Chan DK, Chang KW. GJB2-associated hearing loss: systematic review of worldwide prevalence, genotype, and auditory phenotype. The Laryngoscope. 2014;124(2):E34–53. 10.1002/lary.24332 . 23900770

[26] Tsukada K, Nishio SY, Hattori M, Usami S. Ethnic-Specific Spectrum of GJB2 and SLC26A4 Mutations: Their Origin and a Literature Review. The Annals of otology, rhinology, and laryngology. 2015;124 Suppl 1:61S–76S. 10.1177/0003489415575060 . 25999548

[27] Samanich J, Lowes C, Burk R, Shanske S, Lu J, Shanske A, et al Mutations in GJB2, GJB6, and mitochondrial DNA are rare in African American and Caribbean Hispanic individuals with hearing impairment. American journal of medical genetics Part A. 2007;143A(8):830–8. 10.1002/ajmg.a.31668 . 17357124

[28] Fischer TC, Samanich J, Morrow BE, Chobot-Rodd J, Shanske A, Parikh SR. Genetic evaluation of American minority pediatric cochlear implant recipients. International journal of pediatric otorhinolaryngology. 2009;73(2):195–203. 10.1016/j.ijporl.2008.10.002 . 19081147

[29] Shan J, Chobot-Rodd J, Castellanos R, Babcock M, Shanske A, Parikh SR, et al GJB2 mutation spectrum in 209 hearing impaired individuals of predominantly Caribbean Hispanic and African descent. International journal of pediatric otorhinolaryngology. 2010;74(6):611–8. 10.1016/j.ijporl.2010.03.004 . 20381175

[30] Riazuddin S, Belyantseva IA, Giese AP, Lee K, Indzhykulian AA, Nandamuri SP, et al Alterations of the CIB2 calcium- and integrin-binding protein cause Usher syndrome type 1J and nonsyndromic deafness DFNB48. Nature genetics. 2012;44(11):1265–71. 10.1038/ng.2426 23023331 PMC3501259

[31] Jensen FC, Girardi AJ, Gilden RV, Koprowski H. Infection of Human and Simian Tissue Cultures with Rous Sarcoma Virus. Proc Natl Acad Sci U S A. 1964;52:53–9. 14192657 10.1073/pnas.52.1.53 PMC300571

[32] Belyantseva IA, Boger ET, Naz S, Frolenkov GI, Sellers JR, Ahmed ZM, et al Myosin-XVa is required for tip localization of whirlin and differential elongation of hair-cell stereocilia. Nat Cell Biol. 2005;7(2):148–56. . 15654330 10.1038/ncb1219

[33] Graham FL, Smiley J, Russell WC, Nairn R. Characteristics of a human cell line transformed by DNA from human adenovirus type 5. J Gen Virol. 1977;36(1):59–74. . 886304 10.1099/0022-1317-36-1-59

[34] Arnold K, Bordoli L, Kopp J, Schwede T. The SWISS-MODEL workspace: a web-based environment for protein structure homology modelling. Bioinformatics. 2006;22(2):195–201. 10.1093/bioinformatics/bti770 . 16301204

[35] Krieger E, Koraimann G, Vriend G. Increasing the precision of comparative models with YASARA NOVA—a self-parameterizing force field. Proteins. 2002;47(3):393–402. . 11948792 10.1002/prot.10104

[36] Hager M, Bigotti MG, Meszaros R, Carmignac V, Holmberg J, Allamand V, et al Cib2 binds integrin alpha7Bbeta1D and is reduced in laminin alpha2 chain-deficient muscular dystrophy. The Journal of biological chemistry. 2008;283(36):24760–9. 10.1074/jbc.M801166200 18611855 PMC3259829

[37] Yamniuk AP, Ishida H, Vogel HJ. The interaction between calcium- and integrin-binding protein 1 and the alphaIIb integrin cytoplasmic domain involves a novel C-terminal displacement mechanism. The Journal of biological chemistry. 2006;281(36):26455–64. . 16825200 10.1074/jbc.M603963200

[38] Phillips KR, Biswas A, Cyr JL. How hair cells hear: the molecular basis of hair-cell mechanotransduction. Current opinion in otolaryngology & head and neck surgery. 2008;16(5):445–51. 10.1097/MOO.0b013e32830f4ac8 . 18797287

[39] Kawashima Y, Geleoc GS, Kurima K, Labay V, Lelli A, Asai Y, et al Mechanotransduction in mouse inner ear hair cells requires transmembrane channel-like genes. The Journal of clinical investigation. 2011;121(12):4796–809. 10.1172/JCI60405 22105175 PMC3223072

[40] Kim KX, Beurg M, Hackney CM, Furness DN, Mahendrasingam S, Fettiplace R. The role of transmembrane channel-like proteins in the operation of hair cell mechanotransducer channels. The Journal of general physiology. 2013;142(5):493–505. 10.1085/jgp.201311068 24127526 PMC3813385

[41] Fettiplace R, Kim KX. The physiology of mechanoelectrical transduction channels in hearing. Physiological reviews. 2014;94(3):951–86. 10.1152/physrev.00038.2013 24987009 PMC4101631

[42] Pepermans E, Petit C. The tip-link molecular complex of the auditory mechano-electrical transduction machinery. Hearing research. 2015 10.1016/j.heares.2015.05.005 . 26049141

[43] Huang H, Bogstie JN, Vogel HJ. Biophysical and structural studies of the human calcium- and integrin-binding protein family: understanding their functional similarities and differences. Biochemistry and cell biology = Biochimie et biologie cellulaire. 2012;90(5):646–56. 10.1139/o2012-021 . 22779914

