# Correction: A Novel C-Terminal CIB2 (Calcium and Integrin Binding Protein 2) Mutation Associated with Non-Syndromic Hearing Loss in a Hispanic Family

**DOI:** 10.1371/journal.pone.0141259

**Published:** 2015-10-16

**Authors:** Kunjan Patel, Arnaud P. Giese, J. M. Grossheim, Rashmi S. Hegde, Maria Delio, Joy Samanich, Saima Riazuddin, Gregory I. Frolenkov, Jinlu Cai, Zubair M. Ahmed, Bernice E. Morrow

The fourth author’s name is spelled incorrectly. The correct name is: Rashmi S. Hegde.
