# Supplementary material for: A Novel C-Terminal CIB2 (Calcium and Integrin Binding Protein 2) Mutation Associated with Non-Syndromic Hearing Loss in a Hispanic Family
Source: PLoS One. 2015 Oct 1;10(10):e0133082. doi: 10.1371/journal.pone.0133082 (PMC4591343; doi:10.1371/journal.pone.0133082)
Supplement: S1 Table — We performed a bioinformatic analysis of the c.556C>T mutation in CIB2 to determine its predicted pathogenicity. We provide the SNP ID number from dbSNP, frequency of occurrence in European and African American populations as well as results from pathogenicity predication software. (DOCX) [file pone.0133082.s001.docx]

| **Gene** | **Mutation** | **SNP ID *** | **EVS MAF** | | **Align GVGD** | **SIFT** | **CADD_phred** | **MutationTaster** | **Grantham Score** |
| --- | --- | --- | --- | --- | --- | --- | --- | --- | --- |
|  |  |  | **Freq. EA** | **Freq. AA** |  |  |  |  |  |
| CIB2 | c.556C>T | rs370359511 | 0/8,586 | 1/4,392 | C0 (GV: 353.86 - GD: 0.00) | Deleterious (score: 0, median: 3.60) |  | Disease Causing | 101 |
| [NM_006383.2](http://databases.lovd.nl/whole_genome/transcripts/03372) | (p.Arg186Trp) |  |  |  |  |  | 36 | (p value: 1) | Moderately physiochemical difference |

S1 Table: In silico analysis of the CIB2: c.556C>T (p.Arg186Trp) mutation.

*SNP ID: This not a validated SNP entry on dbSNP

All information except for CADD score was acquired from Alamut version 2.3

Column definitions:

Gene = gene name

Mutation = position of the mutation within the protein

SNP ID = variant in dbSNP

EVS MAF = Exome Variant Server. It contains variants from the NHLBI Exome Sequencing Project (<http://varianttools.sourceforge.net/Annotation/EVS>). EA = European. AA = African American

Align GVGD = web-based server that describes characteristics of sequences (<http://agvgd.iarc.fr>) such as mutations.

SIFT = Software to predict whether an amino acid substitution affects the protein. Deleterious score of 0 means damaging.

CADD = Combined Annotation Dependent Depletion (http://cadd.gs.washington.edu/info) score is a composite score for whether a mutation is considered as damaging and takes many of the other variant annotation tools into account. A CADD score of 36 is highly damaging. Phred is a quality of base calling score.

Mutation Taster = Software that predicts whether a variant can be disease causing (<http://www.mutationtaster.org/>). A p value of 1 supports a role of the variant in causing a disease.

Grantham Score is a tool as part of the Exome Sequencing Project that examines the physiochemical nature of the particular amino acid substitution. The scores range from 0 to 215. The score of 101 means that the variant moderately affects the protein.
